# Haptoglobin Phenotypes Are Associated with the Postload Glucose and Insulin Levels in Pediatric Obesity

**DOI:** 10.1155/2020/6035138

**Published:** 2020-07-09

**Authors:** Roberta Ricotti, Marilisa De Feudis, Caterina Peri, Marco Corazzari, Giulia Genoni, Mara Giordano, Valentina Mancioppi, Emanuela Agosti, Simonetta Bellone, Flavia Prodam

**Affiliations:** ^1^Department of Health Sciences, University of Piemonte Orientale, Novara 28100, Italy; ^2^Department of Translational Medicine, University of Piemonte Orientale, Novara 28100, Italy; ^3^Interdisciplinary Research Center of Autoimmune and Allergic Diseases, University of Piemonte Orientale, Novara 28100, Italy

## Abstract

**Purpose:**

Haptoglobin (Hp) is a protein involved in the acute-phase reaction of inflammation. Humans have three major phenotypes (Hp1-1, Hp1-2, and Hp2-2). Several studies have shown altered Hp regulation in adults with obesity and metabolic alterations. The Hp2-2 phenotype is associated with a high cardiovascular risk. Our aim was to investigate if Hp levels and the Hp2-2 phenotype are associated with glucose metabolism in pediatric obesity.

**Methods:**

We retrospectively studied 192 participants (92 males and 100 females), aged 4–18 years. Clinical and biochemical data were collected. The Hp phenotype (Hp1-1, Hp1-2, and Hp2-2) was identified through Western immunoblot.

**Results:**

Subjects carrying Hp1-1, Hp1-2, and Hp2-2 phenotypes were 13.6, 50.8, and 35.6%, respectively. Hp serum, fasting glucose, and insulin levels, as well as HOMA-IR, were similar among groups. Postload glucose and insulin levels (as insulin AUC) were progressively higher from the Hp1-1 to Hp2-2 phenotype.

**Conclusion:**

To our knowledge, this is the first study on Hp phenotypes conducted in a pediatric population with obesity. We showed that the presence of Hp2 allele is associated with a worse response of glucose load in terms of both glucose and insulin levels. Thus, the Hp2-2 phenotype could predispose in pediatrics, at the same degree of obesity, to a worse glycemic and insulinemic compensation.

## 1. Introduction

Haptoglobin (Hp) is a tetrameric protein constituted of two *α* and two *β*-chains connected by a disulphide bridge. There are two main alleles at the Hp locus, Hp1, and Hp2 that encode for the same *β*-chain, but for two *α*-chains, they differ in length. The two alleles are inherited in a codominant manner and may combine to originate the three major human phenotypes (Hp1-1, Hp2-2, and Hp1-2) [1].

Hp is involved in the acute-phase reaction of inflammation, and its main activity is to bind to cell-free hemoglobin, preventing oxidative stress and tissue damage [2–4]. Hp is mainly produced by the liver, but also at quite lower concentration in immune cells including monocytes and white adipose tissue [4]. Several findings have shown a direct correlation between Hp levels and obesity in humans [4–7], suggesting that it may represent a new chemokine involved in the complex phenomenon of obesity, chronic inflammation, and cardiovascular risk. Furthermore, animal models of Hp deficiency at high-fat diet develop blunted obesity-associated comorbidities [4, 8].

Interestingly, previous studies suggested that clinical data on total Hp levels should be interpreted with caution because although correlated to obesity and cardiometabolic risk, different metabolic outcomes may be associated to the different Hp phenotypes rather than the total levels. In type 2 diabetes, the Hp2-2 phenotype has been associated with increased disease complications, mainly high myocardial infarction and mortality risk [9–11]. Very recently, a post hoc analysis of the ACCORD study revealed that intensive glucose-lowering therapy was efficacious in preventing incident coronary heart disease and cardiovascular events only in individuals showing the Hp2-2 phenotype. This is because hyperglycemia through several mechanisms leads to an increased amounts of circulating Hp2 : Hb complexes with an increased oxidative activity and paradoxically turns the HDL into a proatherogenic and prothrombotic lipoprotein [12].

To date, only a few studies have analyzed Hp levels in obese children [13–15] or young patients with diabetes [16] but none of them considered the Hp phenotype. The aim of our study was to investigate if the Hp levels and Hp2-2 phenotype were associated with obesity comorbidities and glucose metabolism even in childhood.

## 2. Methods

### 2.1. Study Design and Population

We studied 196 obese children and adolescents, aged 4 to 18 years, referred to the Pediatric Endocrine Service of our hospital (January 2005–December 2016) and included in an observational protocol on childhood obesity approved by the Local Ethics Committee (CE 95/12) and was conformed to the guidelines of the European Convention of Human Rights and Biomedicine for Research in Children. Subjects included should satisfy all the following inclusion criteria: (1) obese, according to the International Obesity Task Force (IOTF) body mass index criteria [17]; (2) not on a weight-loss diet; (3) biochemical evaluations including also a 2-hour oral glucose tolerance test (OGTT) had been performed; (4) plasma samples to evaluate the Hp phenotype were available. Exclusion criteria were specific causes of endocrine or genetic obesity, type 1 diabetes, and type 2 diabetes treated pharmacologically, previous kidney or hepatic diseases, and use of specific drugs (oral hypoglycemic agents or antihypertensives). Informed written consent was obtained from all parents before the evaluations after careful explanations were given to each patient.

Subjects with prediabetes in medical nutrition therapy were included, while subjects with type 2 diabetes treated with oral hypoglycemic were excluded. No diagnosis of type 2 diabetes was done.

### 2.2. Anthropometric and Biochemical Measurements

Height was measured to the nearest 0.1 cm using a Harpenden stadiometer, and body weight with light clothing to the nearest 0.1 kg using a manual weighing scale. The BMI was calculated as body weight divided by squared height (kg/m^2^). The BMI standard deviation score (BMISDS) was calculated by the least median squares (LMS) method. Waist circumference (WC) was measured at the high point of the iliac crest around the abdomen and was recorded to the nearest 0.1 cm according to standard methods [18]. The waist-to-height ratio was calculated. Pubertal stages were determined by physical examination, using the criteria of Tanner [19]. Systolic (SBP) and diastolic blood pressure (DBP) were measured three times at 2-minute intervals using a standard sphygmomanometer with an appropriate cuff size after participants were seated quietly for at least 15 minutes according to standards [20] and at least 30 minutes after blood sampling. Mean values were used for the analyses.

After a 12 h overnight fast, blood samples were taken for measurement of the following: glucose (mg/dL), insulin (*μ*UI/mL), total cholesterol (mg/dL), HDL-cholesterol (mg/dL), and triglycerides (mg/dL), using standardized methods in the hospital's laboratory previously described [21]. LDL-cholesterol was calculated by the Friedewald formula. Uric acid (mg/dL) was measured by Fossati method reaction using uricase with a Trinder-like endpoint. Serum Hp levels (mg/ml) were measured by immunonephelometry, using the N antisera antihuman haptoglobin as a reagent (Siemens Healthcare Diagnostics, Deerfield, IL, USA), with a sensibility of 0.26–8.3 g/L and coefficient of variation intercycle of 2.3%, intracycle of 3.5%, and total of 3.9%. Subjects also underwent an OGTT (1.75 g of glucose solution per kg, maximum 75 g), and samples were drawn for the determination of glucose and insulin every 30 min. The area under the curve (AUC) was calculated according to the trapezoidal rule. Insulin resistance was calculated using the formula of homeostasis model assessment (HOMA)-IR. Insulin sensitivity at fasting and during OGTT was calculated as the formula of the Quantitative Insulin-Sensitivity Check Index (QUICKI) and Matsuda Index (ISI) as previously reported [22]. The stimulus for insulin secretion in the increment in plasma glucose as the insulinogenic index was calculated as the ratio of the changes in insulin and glucose concentration from 0 to 30 min (InsI). Glucose was expressed in mg/dL (1 mg/dL = 0.05551 mmol/L) and insulin in *μ*UI/mL (1 *μ*UI/mL = 7.175 pmol/L) in each formula.

### 2.3. Hp Phenotype Identification by Western Immunoblot

**Figure 1:**
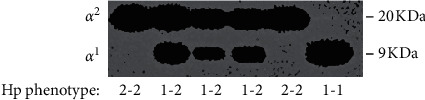
Hp-phenotypization by Western immunoblot in plasma samples of obese pediatric subjects. Diluted plasma samples from six representative obese pediatric subjects were analyzed by WIB under reduced condition to identify the Hp phenotype using a specific antibody against the *α*-chain. The band ∼9 kDa corresponds to the *α*^1^-chain, whereas the band at ∼20 kDa corresponds to the *α*^2^-chain. In the presence of only one band, 20 kDa or 9 kDa, the phenotype was, respectively, 2-2 and 1-1; when both bands were detectable, the phenotype was heterozygous 1-2.

Plasma samples of patients collected by two consequential centrifugations (1300 rpm for 10′ and 2400 rcf for 25′) were diluted 1 : 10 in Dulbecco's phosphate-buffered saline (Sigma-Aldrich, St. Louis, MO) and then loaded in equal amounts in 15% SDS-PAGE gels under reduced conditions. After electrophoretic transfer, the immunoblot polyvinylidene difluoride (PVDF) membranes (Bio-Rad, Hercules, CA) were blocked in Tris-buffered saline 0.1% Tween-20 containing 5% nonfat milk for 1h, then incubated with a primary antibody of Hp *α* (Sigma-Aldrich) diluted 1 : 5000, and detected with horseradish peroxidase-conjugated secondary antimouse IgG diluted 1 : 5000 (Merck Millipore, Darmstadt, Germany). Immunoreactive proteins were detected using enhanced chemiluminescence (Pierce Biotechnology Inc., Rockford, IL, USA) with image capture performed using CCD-camera linked to ChemiDoc (Bio-Rad). In the current study, the Hp phenotype was defined by the presence of *α*-chain bands at either ∼9 kDa (*α*1 : Hp1-1), ∼20 kDa (*α*2 : Hp2-2), or both (Hp1-2).  Figure 1 shows the representative of a Western immunoblot.

### 2.4. Definitions

Subjects were classified as obese according to age- and sex-specific IOTF cutoffs [17]. According to the criteria of Marshall and Tanner [19], the pubertal stage was defined in the presence of a testicular volume of 4 mL for males and the breast at stage 2 for females. SBP and DBP values were evaluated according to percentiles for age, sex, and height, of the National High Blood Pressure Education Program (NHBPEP) Working Group [20]. Arterial hypertension was defined according to the following criteria: (1) > 95^th^ percentile as suggested by the (NHBPEP) Working Group of American Academy of Pediatrics (AAP) [20]. Hypertension was determined if BP values recorded on both the enrollment day and blood sample day were elevated.

Dyslipidemia was considered if hypertriglyceridemia as triglycerides ≥150 mg/dL or reduced HDL-cholesterol levels as HDL-cholesterol ≤40 mg/dL as suggested by IDF (the International Diabetes Federation) criteria for metabolic syndrome classification in children [23]. Impaired fasting glucose (IFG) and impaired glucose tolerance (IGT) were defined by a fasting plasma glucose level of ≥5.6 to 6.9 mmol/L (100–125 mg/dL) and, as 2 h post-OGTT, the glucose level of ≥7.8 to 11.0 mmol/L (140–199 mg/dL), respectively, according to the American Diabetes Association [24].

### 2.5. Statistical Analysis

All data are expressed as mean ± SD, absolute values, or percentages. A sample of 24 individuals for each of three Hp phenotypes has been estimated to be enough to demonstrate a difference of 20 mg/dl in fasting or stimulated glucose levels with a 90.0% of power and a precision of 5.0% according to recently published data on obese children [25, 26]. Due to the lower prevalence of Hp1 genotype in Caucasian and American subjects, the frequency of the Hp1-1 phenotype was considered and the population size was increased at 63 individuals in each group [27]. Distributions of continuous variables were examined for skewness and were logarithmically transformed as appropriate. The differences between genders and pubertal stages were analyzed with the Student *t*-test. ANOVA was used to determine the differences between Hp phenotypes. The ANOVA analysis was also carried out with the following covariates: age, sex, Tanner's stage (Model 1), and BMI in addition to Model 1 (Model 2). Statistical significance was assumed at *p* < 0.05. The statistical analysis was performed with IBM SPSS Statistics for Windows version 22.0 (Chicago, IL, USA). Linkage disequilibrium calculation and haplotype frequency determination was performed with the Haploview software (the Center for Human Genetic Research, Massachusetts General Hospital, and the Broad Institute of Harvard & MIT).

## 3. Results

Of the 196 included subjects, 4 were excluded, 2 because the plasma samples were incorrectly conserved, and 2 because both Hp levels and phenotypes were not detected, and the number was not enough to be compared to the other subjects.

The final dataset included 192 participants (92 males and 100 females), aged 4–18 years, with an age of 11.5 ± 2.8 years. Of those subjects, 60 (31.4%) were prepubertal and 132 (68.6%) were pubertal. The distribution of the three Hp phenotypes was 13.6, 50.8, and 35.6% for Hp1-1, 1-2, and 2-2, respectively, which was in Hardy–Weinberg equilibrium. Clinical and biochemical characteristics of the sample are summarized in Table 1, and HP phenotype comparison is shown in Table 2 considering each phenotype.

Of subjects with hypertriglyceridemia, 1 subject (7.0%) had Hp1-1, 10 subjects (72.0%) had Hp1-2, and 3 (21.0%) had Hp2-2 phenotype. Of subjects with reduced HDL-cholesterol levels, 10 subjects (11.0%) had Hp1-1, 47 subjects (50.0%) had Hp1-2, and 37 subjects (39.0%) had Hp2-2 phenotype. Of subjects with IFG, 2 subjects (12.0%) had Hp1-1, 6 subjects (38.0%) had Hp1-2, and 8 subjects (50.0%) had Hp2-2 phenotype. Of those with IGT, 2 subjects (12.0%) had Hp1-1, 6 subjects (38.0%) had Hp1-2, and 8 subjects (50%) had Hp2-2 phenotype. Nobody had type 2 diabetes. The prevalence of each alteration was not different between prepubertal and pubertal subjects.

Of those subjects with the Hp1-1 phenotype, 1 subject (4.0%) had hypertriglyceridemia, 10 subjects (38%) had reduced HDL-cholesterol levels, and 8 subjects (16.0%) had either IFG or IGT. Of carriers of the Hp1-2 phenotype, 10 subjects (10.0%) had hypertriglyceridemia, 47 subjects (48.0%) had reduced HDL-cholesterol levels, and 12 subjects (12%) had either IFG or IGT. Of subjects with the Hp2-2 phenotype, 3 subjects (4.0%) had hypertriglyceridemia, 37 subjects (54.0%) had reduced HDL-cholesterol levels, and 16 subjects (24%) had either IFG or IGT (Table 2).

HDL-cholesterol levels (*p* < 0.007), ISI index (*p* < 0.008), and QUICKI (*p* < 0.0001) were higher, while fasting glucose levels (*p* < 0.02), fasting (*p* < 0.0001) and post-OGTT insulin levels (120 minutes; *p* < 0.01), and HOMA-IR (*p* < 0.0001) were lower in prepubertal than pubertal subjects.

**Figure 2:**
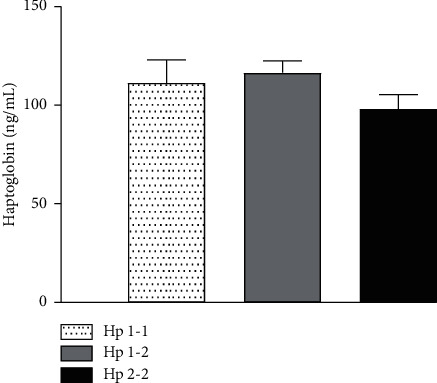
Levels of haptoglobin in the Hp1-1, Hp1-2, and Hp2-2 phenotypes. Data are expressed as mean ± SD. Phenotype 1-1: speckled line and bar; phenotype 1-2: grey line and bar; phenotype 2-2: black line and bar.

**Figure 3:**
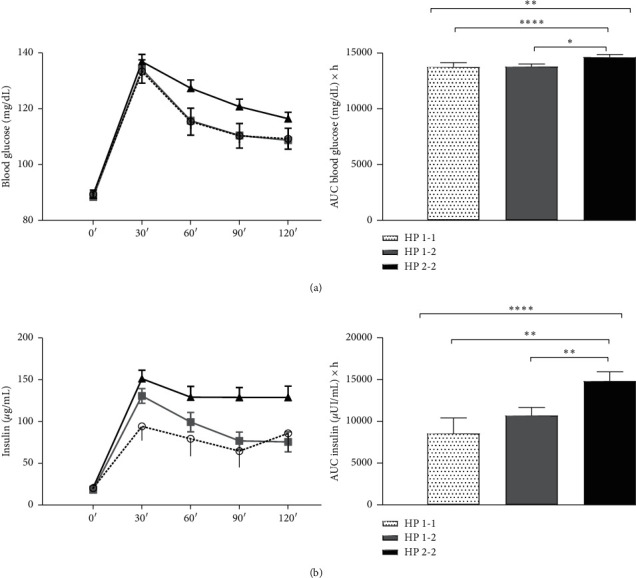
Postload glucose and insulin levels among Hp phenotypes. (a) Glucose levels (mg/dL) at each time point and as AUC (mg/dL × h) at OGTT among Hp phenotypes. (b) Insulin levels (mg/dL) at each time point and as AUC (mg/dL × h) at OGTT among Hp phenotypes. Data are expressed as mean ± SD. AUC = area under the curve. Phenotype 1-1: speckled line and bar; phenotype 1-2: grey line and bar; phenotype 2-2: black line and bar. ^*∗*^*p* < 0.01; ^*∗∗*^*p* < 0.02; ^*∗∗∗∗*^*p* < 0.04; ^*∗∗∗∗∗*^*p* < 0.04.

All subjects were evaluated depending on the Hp phenotype. Hp blood concentrations were similar among the three phenotypes (Figure 2). Fasting blood glucose levels overlapped among them; meanwhile, postload glucose levels progressively increased from the Hp1-1 to Hp2-2 phenotype (time points: 60′*p* < 0.009; 90′*p* < 0.01; 120′*p* < 0.03. Significances were not modified by covariates (Model 2, time points: 60′*p* < 0.03; 90′*p* < 0.01; 120′*p* < 0.05). Consensually, the blood glucose AUC had the same trend (*p* < 0.02) (Figure 3). Similar to blood glucose levels, postload insulin levels were progressively higher from Hp1-1 to Hp2-2 (time points: 30′*p* < 0.04; 60′*p* < 0.05; 90′*p* < 0.02; 120′*p* < 0.05), and significances were not modified by covariates (Model 2, time points: 60′*p* < 0.01; 90′*p* < 0.002; 120′*p* < 0.05). The insulin AUC (330.2 ± 70.5 vs 400.4 ± 38.3 vs 550.9 ± 42.6 *μ*UI/mL; *p* < 0.009) and the sum of insulin levels (*p* < 0.02) were progressively higher from Hp1-1 to Hp2-2 (Figure 3). The HOMA-IR, QUICKI, ISI, and insulinogenic index were similar among Hp phenotypes.

## 4. Discussion

A series of studies have shown higher Hp levels in adult subjects with obesity and metabolic alterations carrying the Hp2-2 phenotype. We demonstrated higher postload glucose and insulin levels in obese children and adolescents carrying the Hp2-2 than Hp1-1 and Hp1-2 phenotypes. To our knowledge, this is the first study on Hp phenotypes conducted in a pediatric population with obesity.

Hp is an acute-phase protein mainly produced by the liver, as previously introduced, and is also a player in oxidative stress [4, 28]. However, our interest in the Hp role in glucose metabolism derives by several reports on other functions of this molecule, including its involvement in vascular complication in type 2 diabetes [27]. Mainly, it is involved in the development of insulin resistance and accumulation of visceral adipose tissue [4, 6, 8, 13, 28]. Hp-null mice had better insulin sensitivity due to modifications of the insulin signal cascade in visceral adipocytes, hepatocytes, and muscle cells. Moreover, Hp-null mice also had adipocytes of reduced size and number, highlighting the role of Hp in the regulation of adipose tissue depots [4].

First of all, the distribution of Hp phenotypes in our study was similar to other published reports on wide cohorts [12, 29–31], suggesting that the domination in some populations of Hp2 had provided some selective advantage [27]. Although some authors reported low Hp serum levels in subjects carrying the Hp2-2 phenotype, we failed to show any difference among the three phenotypes in agreement with other studies [7, 26]. Thus, although we cannot exclude that the population size was not enough to demonstrate subtle different concentrations, the metabolic responses of the subjects seem to be more correlated to the Hp phenotype than to the protein blood concentration. The discrepancy among the studies on the role of Hp circulating levels probably depends on the fact that the phenotype of Hp has not often been previously considered [13–15, 32].

We observed higher stimulated insulin levels from the Hp1-1 to Hp2-2 phenotype. Some data in the literature show that Hp levels are directly correlated with obesity [4, 6, 33] and insulin levels more than insulin resistance [6, 34]. Authors suggested that Hp is a marker of hyperinsulinemia and that insulin could modulate Hp production by the liver and white adipose tissue or both [4, 6]. It has been noted that several authors failed to observe an association between insulin and Hp concentrations in Africans but not in West Americans, and this could derive by the lack of the investigation of the Hp phenotype [33, 34]. In mice with Hp deficiency, insulin signaling is affected in those cells that overexpress Hp if obesity is present, in particular, visceral adipose tissue, and to a much lesser extent the liver and muscle. These animals exhibit a higher response to insulin stimulus [4, 8]. Besides this, insulin signaling could be influenced not only by Hp deficiency but also by the phenotype. Since exon 3 contains the Hp-multimerization domain, the valences of two alleles are different: Hp1 is monovalent while Hp2 is bivalent. This difference influences the protein's structure (dimers in Hp1-1 subjects, linear polymers in Hp1-2 subjects, and cyclic polymers in Hp2-2 subjects) and affects the oxidation capability [2, 12, 35]. Because Hp1-1 has more anti-inflammatory properties, Hp1-1 could promote a reduction in macrophage infiltrates and proinflammatory cytokines in white adipose tissue, resulting in improving the insulin pathway. This hypothesis is in line with findings in obese Hp-deficient models [4].

Moreover, we have also shown that the Hp phenotype is important for the metabolic function of the protein and is associated with a better postload glucose response in subjects carrying Hp1-1, while Hp2 is associated with a worse trend in glucose metabolism in regards of the genetic impact. In humans, several observational and longitudinal studies have established that the Hp2-2 phenotype is an independent risk factor for cardiovascular diseases in adults with type 2 diabetes, mainly in those with higher glucose levels [9, 11, 12]. This phenomenon seems dependent on its lower antioxidant efficacy, more pronounced by glycosylation of the hemoglobin [31, 36, 37]. Because we showed both higher glucose and insulin-stimulated levels in subjects carrying the Hp2-2 phenotype, we can hypothesize that hyperinsulinemia is secondary to subtle peripheral insulin resistance in feeding conditions. This hypothesis is supported by recent findings. Free hemoglobin and HbA1c are bound by Hp forming a complex that is cleared by CD163, a scavenger receptor that is also a novel biomarker of adipose tissue macrophage activation. Expression of CD163 is reduced in Hp2-2 carriers and is also further impaired in condition of hyperglycemia [12, 38]. A recent study observed that CD163 is inversely associated with insulin sensitivity and beta-cell function at OGTT and the risk of dysglycemia in adults at risk for type 2 diabetes [39]. Indeed, higher inflammation and lower oxidative capacity could have a role in altered glucose metabolism, mainly if obesity is present.

Our findings could have a role in clinical practice. Obesity in the pediatric age increases the risk of the incidence of type 2 diabetes in early adulthood in both sexes [40]. Moreover, some children and adults have metabolic healthy obesity and several hypotheses have been suggested; none of these were conclusive [41, 42]. The Hp2-2 phenotype could be one of the mechanisms linking inflammation, insulin resistance, and adipocyte biology with a role in the deterioration of glucose tolerance. Since it does not change with time and specific conditions together with the easy and relatively inexpensive determination, the Hp phenotype could be a promising marker to select young obese patients for a tight follow-up. Studies are also needed to understand if treatments with insulin-sensitizing agents, such as metformin, could determine a different response in insulin resistance based upon Hp phenotypes.

In conclusion, for the first time, we demonstrated higher postload glucose and insulin levels in obese children and adolescents carrying the Hp2-2 phenotype than those carrying the other two Hp phenotypes. Thus, it seems that the Hp2-2 phenotype predisposes in pediatrics, at the same degree of obesity, to a worse glycemic compensation. Further studies are needed to understand if the worst stimulated glucose and insulin levels in Hp2-2 obese children are suggestive of earlier development of altered glucose metabolism. Moreover, studies on long-term metabolic complications of pediatric obesity are required to evaluate if the Hp phenotype is a good biomarker to stratify those at high cardiovascular risk.

## Figures and Tables

**Table 1:** Clinical and biochemical characteristics of the sample.

	All	Prepubertal	Pubertal	*p* value
Subjects	192	60	132	ns
Sex (M/F)	92/100	33/27	59/73	ns
Age (years)	11.5 ± 2.8	8.6 ± 1.8	12.8 ± 2.2	<0.0001
Height (cm)	150.2 ± 14.7	135.0 ± 10.8	157.2 ± 10.12	ns
Weight (kg)	65.1 ± 21.5	49.5 ± 13.4	76.9 ± 16.6	<0.0001
BMI (kg/m^2^)	29.6 ± 4.8	26.8 ± 3.5	30.9 ± 4.8	<0.0001
BMI z-score (kg/m^2^)	2.2 ± 0.5	2.1 ± 0.4	2.3 ± 0.6	ns
Waist circumference (cm)	92.4 ± 13.1	82.9 ± 10.6	96.8 ± 11.8	<0.0001
SBP (mmHg)	126.9 ± 18.4	119.3 ± 14.3	130.4 ± 19.0	<0.0001
DBP (mmHg)	82.0 ± 11.3	77.5 ± 10.5	84.1 ± 11.1	<0.0001
Total cholesterol (mg/dL)	142.2 ± 25.1	145.2 ± 26.7	140.8 ± 24.3	ns
LDL-c (mg/dL)	84.2 ± 21.6	86.0 ± 22.8	83.5 ± 21.0	ns
HDL-c (mg/dL)	41.2 ± 9.7	43.2 ± 9.6	40.3 ± 9.6	<0.01
HDL-cholesterol ≤ 40 (mg/dL)	92 (47.9%)	25 (13.0%)	67 (34.9%)	ns
Triglycerides (mg/dL)	83.2 ± 44.8	77.5 ± 39.4	85.8 ± 47.0	ns
Triglycerides ≥ 150 (mg/dL)	14 (7.3%)	4 (2.1%)	10 (5.2%)	ns
GlcT0' (mg/dL)	88.8 ± 7.2	87.2 ± 7.5	89.6 ± 7.0	<0.02
GlcT120' (mg/dL)	111.8 ± 18.9	108.4 ± 14.1	113.2 ± 20.5	ns
InsT0' (mUI/L)	19.2 ± 12.5	15.2 ± 10.0	21.0 ± 13.1	<0.0001
InsT120' (mUI/L)	96.4 ± 110.9	80.6 ± 114.5	103.0 ± 109.7	<0.01
IFG	14 (7.3%)	3 (1.5%)	11 (5.8%)	ns
IGT	16 (8.3%)	1 (0.5%)	15 (7.8%)	<0.02
HOMA-IR	4.3 ± 2.9	3.3 ± 2.2	4.7 ± 3.1	<0.0001
ISI	3.72 ± 2.99	4.35 ± 3.00	3.47 ± 2.98	<0.01
QUICKI	0.323 ± 0.043	0.332 ± 0.033	0.318 ± 0.046	<0.0001
Haptoglobin (mg/dL)	104.8 ± 41.5	106.4 ± 33.4	104.1 ± 44.4	ns
Hp phenotype 1-1	26 (13.6%)	10 (38.5%)	16 (61.5%)	ns
Hp phenotype 1-2	97 (50.8%)	31 (32.0%)	66 (68.0%)	
Hp phenotype 2-2	68 (35.6%)	19 (27.9%)	49 (72.1%)	

All data are expressed as mean ± SD (standard deviation) or percentage. BMI: body mass index; DBP: diastolic blood pressure; F: female; GlcT0': fasting glucose; HDL-c: high-density lipoprotein-cholesterol; HOMA-IR: homeostatic model assessment of insulin resistance; IFG: impaired fasting glucose; IGT: impaired glucose tolerance; InsT0': fasting insulin; ISI: insulin sensitivity index; Hp: haptoglobin; LDL-c: low-density lipoprotein-cholesterol; M: male; ns: not significant; QUICKI: Quantitative Insulin-Sensitivity Check Index; SBP: systolic blood pressure.

**Table 2:** Clinical and biochemical characteristics according to each HP phenotype.

	Hp1-1	Hp1-2	Hp2-2	*p* value
Subjects	26	97	68	ns
Sex (M/F)	11/15	50/47	30/38	ns
Age (years)	10.7 ± 2.7	11.4 ± 2.8	11.8 ± 2.8	ns
P/PP	16/10	66/31	49/19	ns
Height (cm)	151.0 ± 16.2	150.0 ± 14.8	150.9 ± 14.0	ns
Weight (kg)	70.3 ± 24.6	66.7 ± 16.6	69.9 ± 19.4	ns
BMI (kg/m^2^)	29.9 ± 5.4	28.9 ± 4.3	30.1 ± 4.6	ns
BMI z-score (kg/m^2^)	2.3 ± 0.5	2.3 ± 0.4	2.2 ± 0.5	ns
Waist circumference (cm)	87.4 ± 16.6	90.5 ± 12.9	94.1 ± 13.0	ns
SBP (mmHg)	128.1 ± 15.2	125.3 ± 18.3	128.2 ± 20.1	ns
DBP (mmHg)	82.2 ± 10.7	82.2 ± 10.4	80.6 ± 12.5	ns
Total cholesterol (mg/dL)	141.5 ± 27.6	143.1 ± 25.4	143.4 ± 25.3	ns
LDL-c (mg/dL)	82.7 ± 22.9	84.4 ± 23.8	85.9 ± 21.6	ns
HDL-c (mg/dL)	43.1 ± 7.1	42.2 ± 10.1	40.7 ± 7.8	ns
Triglycerides (mg/dL)	78.3 ± 36.5	77.2 ± 43.0	82.7 ± 40.7	ns
GlcT0' (mg/dL)	89.4 ± 1.5	88.4 ± 0.7	89.2 ± 0.1	ns
GlcT30' (mg/dL)	133.4 ± 4.2	134.2 ± 2.3	136.9 ± 2.6	ns
GlcT60' (mg/dL)	115.4 ± 4.9	115.8 ± 2.7	127.4 ± 3.0	*p* < 0.009
GlcT90' (mg/dL)	110.3 ± 4.4	110.4 ± 2.4	120.8 ± 2.7	*p* < 0.01
GlcT120' (mg/dL)	109.3 ± 3.7	108.7 ± 2.1	116.5 ± 2.3	*p* < 0.03
InsT0' (mUI/L)	20.2 ± 2.5	17.8 ± 1.3	20.7 ± 1.5	ns
InsT30' (mUI/L)	94.0 ± 17.1	130.5 ± 8.8	151.1 ± 10.1	*p* < 0.04
InsT60' (mUI/L)	79.1 ± 20.8	99.1 ± 11.7	128.9 ± 13.0	*p* < 0.05
InsT90' (mUI/L)	76.1 ± 55.4	76.7 ± 10.5	128.7 ± 11.8	*p* < 0.02
InsT120' (mUI/L)	64.4 ± 19.5	75.4 ± 12.0	128.7 ± 13.5	*p* < 0.05
HOMA-IR	4.5 ± 3.0	4.3 ± 2.9	4.6 ± 3.3	ns
ISI	2.80 ± 1.22	2.53 ± 0.80	2.91 ± 2.60	ns
QUICKI	0.321 ± 0.021	0.312 ± 0.030	0.311 ± 0.052	ns

All data are expressed as mean ± SD (standard deviation). Significance is relative to the trend. BMI: body mass index; DBP: diastolic blood pressure; F: female; GlcT0': fasting glucose; HDL-c: high-density lipoprotein-cholesterol; HOMA-IR: homeostatic model assessment of insulin resistance; IFG: impaired fasting glucose; IGT: impaired glucose tolerance; InsT0': fasting insulin; ISI: insulin sensitivity index; Hp: haptoglobin; LDL-c: low-density lipoprotein-cholesterol; M: male; ns: not significant; P: pubertal; PP: prepubertal; QUICKI: Quantitative Insulin-Sensitivity Check Index; SBP: systolic blood pressure.

## Data Availability

Data are available on request through the corresponding author (Flavia Prodam, flavia.prodam@med.uniupo.it).
